# Silencing of Wwox Increases Nuclear Import of Dvl proteins in Head and Neck Cancer

**DOI:** 10.7150/jca.40840

**Published:** 2020-04-06

**Authors:** Asuman Celebi, Ceren Orhan, Betul Seyhan, Nur Buyru

**Affiliations:** Department of Medical Biology and Genetics, Istanbul University-Cerrahpasa, Cerrahpasa Medical Faculty, Istanbul, Turkey

**Keywords:** WWOX, DVL, Wnt/β-catenin pathway, HNSCC, Gene silencing

## Abstract

**Background**: Wnt signaling pathway is associated with a variety of human cancers, including HNSCC. Wnt proteins control cellular events such as proliferation, fate specification, polarity, and migration by transducing signals to the nucleus through several cytoplasmic relay proteins. Although activation of the Wnt/β-catenin pathway is a frequent event in various cancers, there is limited knowledge on the contribution of this signaling mechanism in HNSCC. The Wwox tumor suppressor protein participates in the regulation of Wnt signaling by interacting with Dvl proteins.

**Methods**: In this study, we used qRT-PCR and western blotting to examine the mRNA and protein levels of the Dvls in association with WWOX in HNSCC cell lines and tumor tissues.

**Results**: We found that silencing of WWOX leads to increased nuclear localization of the Dvl proteins in cell lines. However, we detected an increase only in the nuclear localization of Dvl-1 in tumor tissues.

**Conclusions**: Our results suggest that aberrant WWOX expression contributes to HNSCC through the Wnt signaling pathway. Decreased expression of WWOX may function in HNSCC progression by allowing the nuclear localization of Dvl proteins.

## Introduction

HNSCC (Head and Neck Squamous Cell Carcinoma) is the sixth most prevalent cancer with a high mortality rate worldwide 1. Despite the efforts of scientists to understand the molecular mechanism of HNSCC, the 5-year survival is still 50-60% 2. Such a high mortality rate is due to the lack of suitable markers for early detection of disease and failure in response to therapeutic agents. Therefore, understanding the complex mechanism of the disease will lead to early diagnosis and help to develop novel effective therapeutics.

Different molecular mechanisms and pathways are involved in the development of HNSCC including the Wnt signaling pathway which regulates cell proliferation, differentiation and cell motility through the canonical (Wnt/β-catenin) and non-canonical pathways 3, 4. In the canonical pathway binding of Wnt proteins to Frizzled (Fzd) receptors recruit the Dishvelled (Dvl) proteins to the plasma membrane. This leads to activation of the Dvl proteins through phosphorylation. Activated Dvl proteins inactivate the β-catenin degradation complex (APC- Axin- GSK3β) by binding to Axin. As a result, β-catenin gradually increases in the cytosol and migrates into the nucleus where it activates transcription of the target genes 5.

While activation of the Wnt/β-catenin pathway is a frequent event in various cancers, there is limited knowledge on the contribution of this signaling mechanism in HNSCC 6-8. Although Wnt and its receptors Fzd have been analyzed in HNSCC the intracellular components of the pathway have not been investigated 9-11. There are three Dvl protein coding genes in the human genome. These proteins work as down-stream effectors of the Fzd proteins but up-stream of β-catenin in the Wnt signaling pathway 5. Several binding partners of the Dvl proteins have been identified which act in the Wnt/β-catenin pathway. Recently, Bouteille et al. 12 reported that the WWOX protein interacts with Dvl proteins and inhibits the Wnt/β-catenin pathway by preventing the nuclear import of these proteins.

The WWOX gene is located at a common fragile site (FRA16D) which is frequently involved in human cancers. In addition to the homozygous deletion of the WWOX gene its decreased or aberrant expression has also been reported in multiple human cancers 13-16. In our previous study we have shown that the WWOX gene is inactivated in HNSCC as a result of genetic and epigenetic alterations 16. In the present study we aimed to investigate the association between Wwox and Dvl proteins in human HNSCC tumors and cell lines. This is the first study in the literature investigating the expression levels of Dvl proteins in HNSCC.

## Material and Methods

### Patients

Tumor and matched adjacent non-cancerous tissues diagnosed with HNSCC, from 98 patients undergoing surgery at the Istanbul University Cerrahpasa Medical Faculty, Department of Head and Neck Surgery were studied. All patients were selected from subjects who were operated with the diagnosis of HNSCC according to previous biopsy results. The patients who had previous chemotherapy or radiotherapy were excluded from the study. All of the samples were also HPV-negative. Inclusion criteria were first time diagnosed HNSCC patients over 18 years. This study was approved by the Cerrahpasa Medical Faculty Ethics Committee (Approval Number:83045809/604.01/02-173216). All subjects gave written informed consent in accordance with the Declaration of Helsinki. Clinicopathological characteristics including age, gender, TNM clinical staging, location of tumor, invasion status, pathology and histologic grade are shown in Table 1.

### Cell Culture

HET-1A (CRL-2692) and SCC-15 (CRL-1623) cell lines were purchased from ATCC (American Type Culture Collection, Wesel, Germany) and were cultured in Dulbecco's modified Eagle's Medium (DMEM) supplemented with 400 ng/ml hydrocortisone (H4001, Thermo Fisher Scientific, MA, USA), 10% fetal bovine serum (FBS, 10270106, Thermo Fisher Scientific, MA, USA), 100 U/ml penicillin and 100 µg/ml streptomycin at 37°C with 5% CO_2_.

### Transfection with siRNA

Transient transfection of a non-targeting control (sc-37007, Santa Cruz Biotechnology, TX, USA) and WWOX siRNA (sc-44193, Santa Cruz Biotechnology, TX, USA) into the HET-1A cell lines was performed using the short interfering RNA (siRNA) Reagent System (sc-45064, Santa Cruz Biotechnology, TX, USA) according to the manufacturer's protocol. Cells (4x10^5^ cells/well) were plated in a 6-well plate and grown to 60-70% confluence before transfection. Cells were then transfected with equal amounts of the siRNA (40 pmol) and harvested at 24 hours for RNA and protein analysis.

### RNA and qRT-PCR Analysis

To quantitate expression of the target genes, total RNA was extracted from cell lines and human tumor samples using the PureLink^TM^ RNA Mini Kit (Thermo Fisher Scientific, MA, USA) and 400 ng of total RNA was reverse-transcribed using the RevertAid first-strand cDNA synthesis kit (Thermo Fisher Scientific, MA, USA) in 20 μL reaction volume with random hexamer primers.

Expression levels were quantified by quantitative Real Time PCR (qRT-PCR) using the LightCycler 480 (Roche Diagnostics, Mannheim, Germany). qRT-PCR was performed using TAMRA hydrolysis probes for WWOX and SYBR Green for DVL-1,DVL-2, DVL-3 expression analysis. All values were calculated by normalizing the levels of each target for each cDNA to the Glucose-6-Phosphate Dehydrogenase (G6PD) levels and then by using this value to calculate fold-changes compared to control. Primer sequences which were used for expression analysis are shown in Table 2. qPCR reactions were performed at least for 3 times.

### Western Blot Analysis

Nuclear and cytoplasmic protein lysates were obtained from cell lines and human tissues using the Nuclear Extraction Kit (Chemicon International, CA, USA) according to the manufacturer's protocol. Equal amounts of protein lysates (100 µg) were loaded onto 4-10% SDS-PAGE gels. After electrophoresis, proteins were transferred to nitrocellulose membranes (Thermo Fisher Scientific, MA, USA) using the iBlot® Dry Blotting system (Thermo Fisher Scientific, MA, USA) for Western blot analysis. The membranes were blocked with 5% Bovine Serum Albumine (sc-2323, Santa Cruz Biotechnology, TX, USA)/TBST (sc-24953, Santa Cruz Biotechnology, TX, USA) for 1.5 h at room temperature and incubated overnight at 4°C with the primary antibodies against Wwox (dilution 1:1000, AF6399, R&D System, MN, USA), Dvl1 (dilution 1:700, NBP1-58318, Novus Biologicals, CO, USA), Dvl2 (dilution 1:1000, NBP1-32388, Novus Biologicals, CO, USA), Dvl3 (dilution 1:200, NBP2-24674, Novus Biologicals, CO, USA). After washing with TBST 3 times, the membranes were incubated with HRP-conjugated goat-anti sheep (dilution 1:7000, HAF016, R&D System, MN, USA) and goat-anti rabbit (dilution 1:5000, sc-2004, Santa Cruz Biotechnology, TX, USA) antibodies for 2h at room temperature. Finally, the target proteins were detected using the HRP Chemiluminescent Substrate Reagent Kit (Cat#WP20005, Thermo Fisher Scientific, MA, USA). Quantification of the proteins was performed using the Image J Software (http://rsb.info.nih.gov/ij/download.html).

### Statistical Analyses

Statistical analysis was performed using the SPSS 21.0 (IBM® SPSS® Statistics, IBM Corporation Somers, NY, USA) program. Paired Sample t-Test was used and p<0.05 was considered as statistically significant for data which are normally distributed. Wilcoxon Signed Rank Test, non-parametric counterpart of the Paired Sample t-Test, was used for unequally distributed expression levels.

## Results

**Figure 1:**
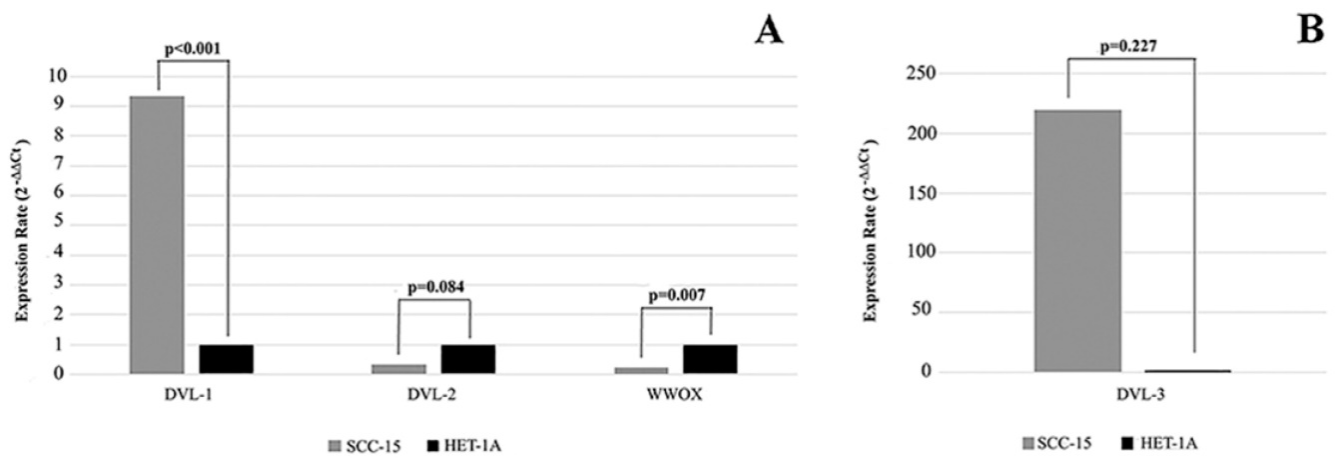
Expression levels of *WWOX, DVL-1, DVL-2* and *DVL-3* in SCC-15 and HET-1A cell lines. **SCC-15:** human tongue squamous cancer cell line; **HET-1A:** human esophageal squamous epithelial cell line.

We first investigated the mRNA and protein levels of WWOX, DVL-1, DVL-2 and DVL-3 in human SCC-15 and HET-1A cell lines. WWOX expression rate was significantly higher in the HET-1A cell line compared to the SCC-15 cell line. As shown in Figure 1 we found that the SCC-15 cell line expresses significantly higher levels of DVL-1 and DVL-3 mRNA compared to the HET-1A cell line, whereas DVL-2 mRNA was down-regulated.

Bouteille et al. 12 have reported that WWOX inhibits the Wnt/β-catenin pathway via interacting with the Dvl proteins. Therefore, to test the effect of the WWOX gene on the intracellular components of the Wnt/β-catenin pathway we silenced the WWOX gene in the high-WWOX expressing HET-1A cell line. After silencing WWOX we investigated the expression levels. Despite silencing of the WWOX gene, expression of DVL-2 was down-regulated in WWOX-siRNA transfected cells compared to cells which were transfected with non-targeting control siRNA (Table 3), but DVL-3 expression was up-regulated.

We also investigated the cytoplasmic/nuclear ratios of the DVL-1, DVL-2 and DVL-3 proteins by Western blotting. We detected high levels of WWOX protein in the cytoplasm of the HET-1A cell line than in the nucleus whereas the protein level was lower in the cytoplasm of the SCC-15 cell line. Cytoplasmic and nuclear levels of the Dvl-1 protein were the same in HET-1A cells although the cytoplasmic levels were 20% higher in the SCC-15 cell line. The Dvl-2 levels were 2-times higher in the cytoplasm of HET-1A cells than in the nuclei whereas the cytoplasmic level was 1.4-times higher in the SCC-15 cell line (Table 4). After silencing the WWOX gene in the HET-1A cell line we observed a decrease in the nuclear localization of the Wwox protein in the cells transfected with WWOX siRNA compared to those transfected with non-targeting control siRNA. In response to silencing of WWOX 10%, 90% and 10% increases in the nuclear localization of the Dvl-1, Dvl-2 and Dvl-3 proteins were observed, respectively.

**Figure 2:**
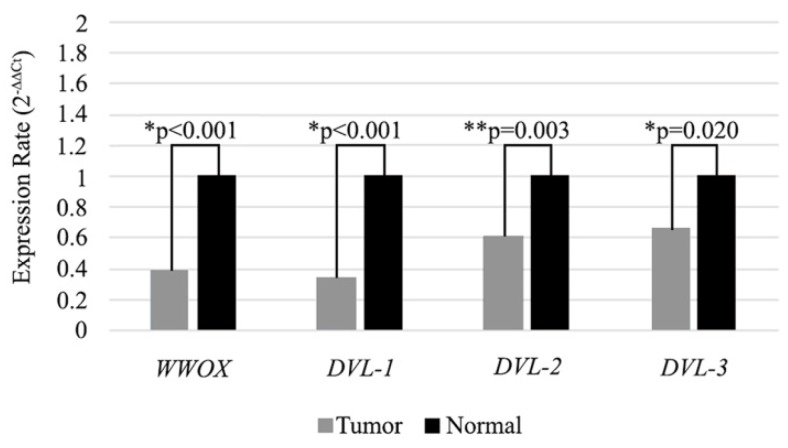
Expression levels of* WWOX*, *DVL-1*, *DVL-2* and *DVL-3* genes in tissue samples. Mean 2^-∆∆CT^ values are shown as a bar graph. *Statistical analysis were performed using Wilcoxon Signed Rank Test. **Statistical analysis were performed using Paired Sample T-Test.

In our previous study we observed significantly low level of WWOX gene expression in HNSCC tumor tissues. In the present study, we also analyzed the expression levels of the WWOX gene in 98 HNSCC tumor tissues and adjacent non-cancerous tissue samples. We detected low mRNA expression in the tumor tissues compared to adjacent non-cancerous tissue samples (Figure 2). In accordance with cell line results expression of the WWOX, DVL-1, DVL-2 and DVL-3 genes were lower (73.5 %, 51%, 56.1% and 50%) in the tumor tissues and the differences were statistically significant (Table 5).

**Figure 3:**
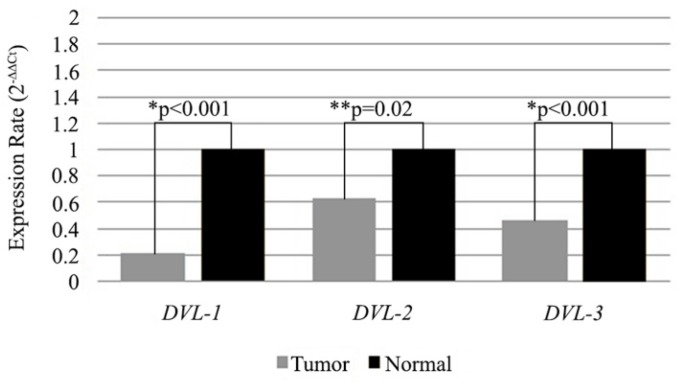
Expression levels of *DVL-1*,* DVL-2* and *DVL-3* genes in *WWOX* down-regulated tissue samples. Mean 2^-∆∆CT^ values are shown as bar graph. *Statistical analysis were performed using Wilcoxon Signed Rank Test. **Statistical analysis were performed using Paired Sample T-Test

Additionally we also compared expression levels of the DVL genes in low WWOX expressing tumor samples with the matched non-cancerous tissues. All of the three genes were low in tumors compared to non-cancerous tissue samples and differences were statistically significant (Figure 3).

**Figure 4:**
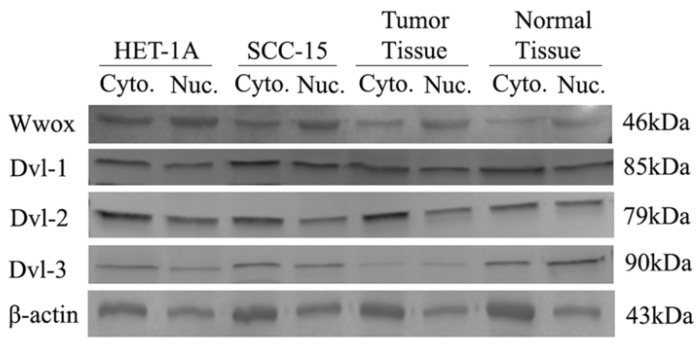
Intracellular distribution of Wwox, Dvl-1, Dvl-2 and Dvl-3 proteins in HET-1A and SCC-15 cell lines.

Subsequently, we also investigated cytoplasmic and nuclear protein levels in a subgroup (n=50) of the tumors and corresponding non-cancerous tumor samples. As a result of this investigation, we identified down-regulation in the cytoplasmic Wwox proteins in tumor tissues compared to non-cancerous tissue samples. While, Dvl-1 protein levels were increased in the nuclei of the tumor tissues, there was a decrease in the nuclear levels of the Dvl-2 and Dvl-3 proteins (Table 6, Figure 4).

## Discussion

Aberrant activation of the Wnt/β-catenin pathway is one of the most common mechanisms in human cancers including HNSCC 6. There are more than 50 known protein components of the canonical Wnt signaling pathway 5. Therefore, abnormalities in any one of several key members of this pathway may contribute to oncogenesis. As a result of GO (Gene Ontology) and KEGG (Kyoto Encyclopedia of Genes and Genomes) pathway analysis Yan et al. 17 reported that genes in the Wnt signaling pathway are upregulated in HNSCC. Furthermore, Rampias et al. 18 reported that activation of the Wnt pathway was associated with the HPV E6/E7 protein in HNSCC. Despite investigation of the components of the Wnt/β-catenin pathway, the importances of Dvls which are central mediators of the pathway are poorly defined in cancers including HNSCC 19. Therefore, in the present study we investigated expression rates of DVL mRNAs in HNSCC tumor tissues and cell lines. The majority of the reports in the literature indicate that Dvls are overexpressed in various tumor types 20-23. However, contradictory reports are also present. For instance, Li et al. 24 reported a decrease in DVL1 and DVL2 expression in contrast to an increase in DVL3 expression in NSCLC. The expression changes of DVL mRNAs available in the ICGC data base 25, which includes results from 981 HNSCC tumors are similar to the data reported by Li et al. In line with this, we detected down-regulation of DVL2 mRNA in HNSCC tumor tissue samples compared to their non-cancerous counterparts. However, the expression profiles were different in cell lines. Expression rates of DVL1 and DVL3 were higher in the SCC-15 tumor cell line compared to the HET-1A normal cell line. This difference may be due to the fact that regulation of gene expression in vivo or under physiological conditions may be distinct from that observed in vitro. The results from databases (Expression Atlas, GTEx and CCLE) are also in line with our results. As seen in those databases WWOX and DVL2 expression rates are low in tongue, oral and pharyngeal squamous cell carcinoma cell lines including the SCC-15 cell line. In contrast to WWOX and DVL2, expression rates of DVL1 and DVL3 were higher in cancer cell lines compared to normal 26-28.

It is well known that Dvls are main intracellular effector molecules of the Wnt/β-catenin pathway. Dvls integrate immerse number of upstream signals by interacting with more than 50 different proteins 29. Additionally, upon activation Dvl proteins shuttle between the cytoplasm and nucleus which is crucial for their functions 19, 30, 31. In 2009 Bouteille et al. 12 reported that the tumor suppressor protein WWOX is a negative regulator of the Wnt/β-catenin pathway and inhibits the transcriptional activity of this pathway by preventing nuclear import of the Dvl proteins. In our previous study we showed that the WWOX gene is inactivated in HNSCC as a result of genetic or epigenetic alterations 16. Here, to investigate the effect of Wwox on the cellular localization of Dvl proteins in HNSCC we silenced the WWOX gene in the HET-1A cell line. As a result of silencing we observed up-regulation in DVL3 gene expression. The cytoplasmic function of Dvl proteins has been investigated in the context of tumorigenesis by previous studies. However, neither their expression nor localization has been investigated in head and neck cancer. Therefore, we also evaluated the subcellular localization of Dvl proteins. Supporting the findings of Bouteille et al. 12 we observed an increase in the nuclear localization of all Dvl proteins when the WWOX gene is silenced in the HET-1A cell line. This indicates that the Wwox protein has an effect on the localization of all three Dvl proteins. Recent studies show that nuclear Dvl proteins bind to the promoters of cell cycle and cell division regulatory genes such as c-Myc and cyclin D1 32. More recently, Castro-Piedros et al. 33 reported that nuclear Dvl exerts its effect in breast cancer via binding to the promoter and regulating transcription of CYP19A1. In human HNSCC tumor tissues we observed only an increase in the nuclear localization of the Dvl-1 protein. Our results suggest that, both the expression rate and cellular localization of Dvl proteins are important players in HNSCC and need to be investigated more deeply. To clarify the effect of WWOX on the expression and cellular localization of Dvl in HNSCC further *in vitro* and *in vivo* studies need to be conducted.

## Conclusion

After the identification of Wwox as a tumor supressor a new interacting partner has been identied regularly. Previously, it has been shown that Wwox exerts its tumor suppressor function via interacting with p73, AP2-γ,C-jun, ErbB-4 and finally Dvl proteins 12, 34-38. In our previous study we reported that WWOX contributes to HNSCC tumor formation or progression as a result of genetic or epigenetic inactivations. Our present findings suggest that Wwox exerts its tumor suppressor function via Dvl proteins which are the key components of the oncogenic Wnt/β-catenin pathway.

## Figures and Tables

**Table 1:** Clinicopathological parameter of the HNSCC patients

Clinicopathological Parameters		Number of Patients n (%)
**Age**	**≤50**	18 (18.4)
	**≤50**	80 (81.6)
**Gender**	**Male**	82 (83.7)
	**Female**	16 (16.3)
**TNM Stage**	**Early (1+2)**	18 (18.4)
	**Advanced (3+4)**	80 (81.6)
**Location of Tumor**	**Larnyx**	63 (64.3)
	**Pharynx**	6 (6.1)
	**Oral Cavity**	21 (21.4)
	**Paranasal**	2 (2)
	**Parotid**	3 (3.1)
	**Unknown**	3 (3.1)
**Pathology**	**SCC**	92 (93.9)
	**Non-SCC**	6 (6.1)
**Histologic Grade**	**Low (1+2)**	43 (43.9)
	**High (3+4)**	41 (41.8)
	**Unknown**	14 (14.3)
**Smoking**	**Yes**	60 (61.2)
	**No**	21 (21.4)
	**Unknown**	17 (17.3)
**Blood Vessel Invasion**	**Present**	33 (33.7)
	**Absent**	44 (44.9)
	**Unknown**	21 (21.4)
**Lymphatic Invasion**	**Present**	58 (59.2)
	**Absent**	19 (19.4)
	**Unknown**	21 (21.4)
**Perineural Invasion**	**Present**	40 (40.8)
	**Absent**	36 (36.7)
	**Unknown**	22 (22.4)
**Cartilage Invasion**	**Present**	39 (39.8)
	**Absent**	21 (21.4)
	**Unknown**	38 (38.8)

**Table 2:** Primers used in qRT-PCR for investigating expression levels.

***WWOX***	F-5'-CACGCGGGGTCACGTCGAA-3'
	R-5'-TGGTGGCAGCTCCCTGTTGC-3'
***DVL-1***	F-5'-CGAAGCTACTTCACCGTCCCA-3'
	R-5'-GCCTCTTCCAGCTCGTAGCG-3'
***DVL-2***	F-5'-ATCCTTCCACCCTAATGTGTCCA-3'
	R-5'-CGCCATGCTCACTGCTGTCT-3'
***DVL-3***	F-5'-TTCACATTGCCCAGGAGCGAG-3'
	R-5'-GGATGGACAAGTGGAAGTCGTCTAG-3'
***G6PD***	F-5'-ATGCCTTCCATCAGTCGGATACA-3'
	R-5'-ATAGCCCACGATGAAGGTGTTTTC-3'

**Table 3:** Mean expression levels of *WWOX, DVL-1, DVL-2* and *DVL-3* in cell lines. HET-1A: Human esophageal squamous epithelial cell line, SCC-15: Human tongue squamous cancer cell line.

Gene	Cell Lines	∆Ct	∆∆Ct	2^-∆∆Ct^
***WWOX***	**HET-1A**	2.44	0	1
	**SCC-15**	4.64	2.2	0.22
	**HET-1A(+Control siRNA)**	1.09	0	1
	**HET-1A(+WWOX siRNA)**	4.89	3.8	0.07
***DVL-1***	**HET-1A**	3.19	0	1
	**SCC-15**	-0.03	-3.22	9.32
	**HET-1A(+Control siRNA)**	2.91	0	1
	**HET-1A(+WWOX siRNA)**	2.94	0.03	0.98
***DVL-2***	**HET-1A**	-0.7	0	1
	**SCC-15**	0.8	1.5	0.35
	**HET-1A(+Control siRNA)**	-1.09	0	1
	**HET-1A(+WWOX siRNA)**	-0.13	0.96	0.51
***DVL-3***	**HET-1A**	7.59	0	1
	**SCC-15**	0.19	-7.78	219.79
	**HET-1A(+Control siRNA)**	6.6	0	1
	**HET-1A(+WWOX siRNA)**	5.23	-1.37	2.6

**Table 4:** Localization rates of Wwox, Dvl-1, Dvl-2, Dvl-3 proteins in HET-1A (Human esophageal squamous epithelial cell line), SCC-15 (Human tongue squamous cancer cell line) and HET-1A cells threated with WWOX siRNA and control siRNA.

Protein	Cytoplasm/Nucleus			
	HET-1A	SCC-15	HET-1A (+Control siRNA)	HET-1A (+WWOX siRNA)
**Wwox**	1.75	0.56	1.04	1.41
**Dvl-1**	1.04	1.21	2.53	2.06
**Dvl-2**	2.01	1.43	2.23	1.14
**Dvl-3**	0.69	0.40	1.01	0.89

**Table 5:** Distribution of the expression changes of *WWOX*,* DVL-1*,* DVL-2* and* DVL-3* in tissue samples

Gene	mRNA Expression		
	Decrease n( %)	No change n( %)	Increase n( %)
***WWOX***	72 (73.5)	2 (2)	24 (24.5)
***DVL-1***	50 (51)	16 (16.3)	32 (32.7)
***DVL-2***	55 (56.1)	7 (7.1)	36 (36.7)
***DVL-3***	49 (50)	11 (11.2)	38 (38.8)

**Table 6:** Cytoplasmic/nuclear ratios of Wwox, Dvl-1, Dvl-2 and Dvl-3 in 50 HNSCC tissues”

Protein	Tissue	Mean	± SD	*p
**Wwox**	Tumor	2.8	2.9	0.822
	Normal	2.7	3.0	
**Dvl-1**	Tumor	2.7	2.2	0.002
	Normal	4.9	5.2	
**Dvl-2**	Tumor	1.91	1.45	0.001
	Normal	1.13	0.74	
**Dvl-3**	Tumor	1.62	0.94	0.002
	Normal	1.07	0.8	

Data are reported as mean and standard deviation. *Statistical analysis were performed using Paired Sample T-Test.

## Abbreviations

DMEM: dullbecco's modified eagle's medium
DVL: dishevelled
FBS: fetal bovine serum
Fzd: frizzled
G6PD: glucose-6-phosphate dehydrogenase
GO: gene ontology
HNSCC: head and neck squamous cell carcinoma
KEGG: kyoto encyclopedia of genes and genomes
ICGC: International Cancer Genome Consortium
GTEx: Genotype Tissue Expression
qRT-PCR: quantitative real time polymerase chain reaction
SD: standard deviation
TBST: tris buffer saline tween 20
WWOX: ww domain containing oxidoreductase
